# Estimation of an Optimal Chemotherapy Utilisation Rate for Upper Gastrointestinal Cancers: Setting an Evidence-Based Benchmark for the Best-Quality Cancer Care

**DOI:** 10.1155/2015/753480

**Published:** 2015-03-26

**Authors:** Weng Ng, Susannah Jacob, Geoff Delaney, Viet Do, Michael Barton

**Affiliations:** ^1^Ingham Institute for Applied Medical Research, Liverpool, Sydney, NSW, Australia; ^2^Collaboration for Cancer Outcomes Research and Evaluation, Sydney, NSW, Australia; ^3^Faculty of Medicine, University of New South Wales, Sydney, NSW, Australia

## Abstract

*Aims*. The proportion of patients with upper gastrointestinal cancers that received chemotherapy varies widely in Australia and internationally, indicating a need for a benchmark rate of chemotherapy utilisation. We developed evidence-based models for upper gastrointestinal cancers to estimate the optimal chemotherapy utilisation rates that can serve as useful benchmarks for measuring and improving the quality of care. *Materials and Methods*. Optimal chemotherapy utilisation models for cancers of the oesophagus, stomach, pancreas, gallbladder, and primary liver were constructed using indications for chemotherapy identified from evidence-based guidelines. *Results*. Based on the best available evidence, the optimal proportion of upper gastrointestinal cancers that should receive chemotherapy at least once during the course of the patients' illness was estimated to be 79% for oesophageal cancer, 83% for gastric cancer, 35% for pancreatic cancer, 80% for gallbladder cancer, and 27% for primary liver cancer. *Conclusions*. The reported chemotherapy utilisation rates for upper gastrointestinal cancers (with the exception of primary liver cancer) appear to be substantially lower than the estimated optimal rates suggesting that chemotherapy may be underutilised. Further studies to elucidate the reasons for the potential underutilisation of chemotherapy in upper gastrointestinal tumours are required to bridge the gap between the ideal and actual practice identified.

## 1. Introduction

One of the most fundamental requirements to the provision of quality cancer care is to ensure that patients receive timely and appropriate treatment following their diagnosis [1]. The Institute of Medicine's National Cancer Policy Board in the United States have concluded in their “Ensuring the Quality of Cancer Care” report that a substantial number of cancer patients were receiving suboptimal treatment and recommended establishment of benchmarks for quality improvements [1]. In addition, the EUROCARE-4 study postulated that some of the survival differences seen in certain tumour groups between the European countries may be related to the variation in the utilisation of treatments such as adjuvant chemotherapy in node-positive breast cancer, as well as the variable application of evidence-based guidelines [2].

Collectively, the upper gastrointestinal cancers represent approximately 7% of all registered cancers in Australia [3]. Major discrepancies between the optimal and actual rates of radiotherapy utilisation for upper gastrointestinal cancers have previously been demonstrated [4]. Several population-based studies have reported large variations in the proportion of patients with upper gastrointestinal cancers who have received chemotherapy, but there are no current benchmarks for comparison [5–16]. In this study, we constructed evidence-based models to estimate the optimal chemotherapy utilisation rates in patients with upper gastrointestinal cancers to serve as a useful benchmark for measuring and improving the quality of care.

## 2. Materials and Methods

### 2.1. Indications for Chemotherapy

An indication for chemotherapy was defined as a clinical situation in which chemotherapy is the treatment of choice on the basis of superior clinical outcomes in comparison to other treatment modalities (including best supportive care or no treatment). The superiority of chemotherapy over other treatment options could be based on survival, quality of life, or toxicity profile. Chemotherapy could be recommended either alone or in combination with radiotherapy or surgery. The list of drugs classified as chemotherapeutic agents were defined according to the SEER^*^RX Database, which is an Interactive Antineoplastic Drug Database developed by the Surveillance, Epidemiology, and End Results Program of the United States National Cancer Institute [17].

The indications for chemotherapy for each cancer site were determined from English language treatment guidelines issued by reputed national and international institutions. These include guidelines from the United States National Comprehensive Cancer Network (NCCN) [18–21] and National Cancer Institute (NCI) [22–26], the Canadian British Columbia Cancer Agency (BCCA) [27] and Cancer Care Ontario (CCO) [28–31], and the Scottish Intercollegiate Guidelines Network (SIGN) [32]. The hierarchy of levels of evidence used to justify the indications for chemotherapy was adapted from the Australian National Health and Medical Research Council (NHMRC) [33]. Based on the best evidence available, we generated a list of clinical scenarios for which chemotherapy in upper gastrointestinal cancers was indicated (see Table 1).

### 2.2. Incidence Data

The data on the proportion of tumour and patient attributes for which chemotherapy was indicated (Table 2) were ranked using a previously published hierarchy [34]. When data on the same attributes were available from multiple sources, the data ranked highest quality were used as the base value in the chemotherapy utilisation tree. In situations where data obtained from multiple sources were ranked of equivalent quality, the larger sample size was chosen.

### 2.3. Performance Status

Patient performance status (PS) is an important prognostic factor which also predicts benefits from treatment and is used in clinical trials and daily practice to select and stratify eligible patients for chemotherapy [54]. Chemotherapy is generally recommended for patients with good performance status (ECOG 0–2) [18]. Unfortunately specific performance status data were available only for pancreatic cancer and not for the other upper gastrointestinal cancers. Therefore we estimated the proportion of age-adjusted good performance status patients from the New South Wales (NSW) Population Health Survey 2005 data on “difficulty doing work” by each of the corresponding age groups [36] and data on the age distribution of upper gastrointestinal cancers in the Australian population [3].

Participants in the NSW Population Health Survey were asked about the degree of difficulty that they had experienced in undertaking daily work or activities (no difficulty, little difficulty, some difficulty, much difficulty, or unable to carry out daily activities or work) in the past 4 weeks. This scale shows reasonable correlation with the Eastern Cooperative Oncology Group (ECOG) [55] scoring scales used to measure performance status (PS). Good performance status (ECOG 0–2) was assumed in those who reported “no difficulty at all,” “a little bit of difficulty,” and “some difficulty.” Participants who reported “much difficulty” or who could not do work or carry out daily activities were assumed to have poor performance status, corresponding to ECOG 3-4. In the NSW Population Health Survey, the rate of good PS (ECOG 0–2) patients varied from 92% (<55 years old) to 87% (>75 years old). The age-adjusted proportion of good PS patients was estimated to be 91% for oesophageal cancer, 89% for gastric cancer, 90% for primary liver cancer, and 68% for gallbladder cancer. As there was some uncertainty whether respondents with “some difficulty” should be included in the good PS group, sensitivity analysis to assess the variation on the estimated optimal utilisation if they were excluded was performed.

### 2.4. Optimal Chemotherapy Utilisation Rate

We merged the indications for chemotherapy treatment in Table 1 and the epidemiological data on the proportions of tumour and patient attributes in Table 2 using the TreeAge Pro 2007 software (version 1.0) to construct the optimal chemotherapy utilisation trees for oesophageal cancer, pancreatic cancer, gastric cancer, gallbladder cancer, and primary liver cancer. In the utilisation tree, each patient with an indication for chemotherapy treatment was only counted once and the tree was terminated at the point of chemotherapy being recommended even if the patient may have subsequent indications during the course of their illness. This was to standardize the comparison of the optimal rate with reported actual rates of chemotherapy utilisation that was defined as the number of patients treated with chemotherapy for the first time divided by the incidence of each specific cancer type during a period.

We calculated the optimal utilisation rates for each cancer site by summing the proportion of patients for each clinical scenario for which chemotherapy was indicated. The utilisation trees were externally reviewed by independent experts to ensure clinical validity. Appropriate changes were made to the models based on the feedback received. The panel of reviewers included members of the Australasian Gastrointestinal Trials Group, New South Wales Oncology Group, and Victoria Cooperative Oncology Group.

### 2.5. Statistical Analysis

We tested the robustness of the chemotherapy utilisation model with univariate sensitivity analyses. Univariate sensitivity analyses were conducted if the incidence of epidemiological data obtained varied by more than 10% or when there were disagreements between guidelines for a chemotherapy treatment indication.

## 3. Results

**Figure 1:**
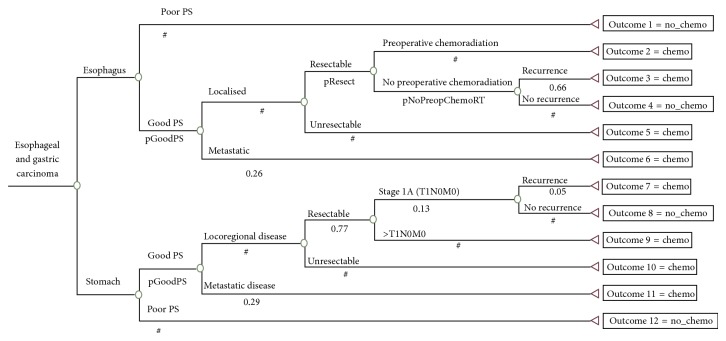
Optimal chemotherapy utilisation tree for oesophageal and gastric cancers.

**Figure 2:**
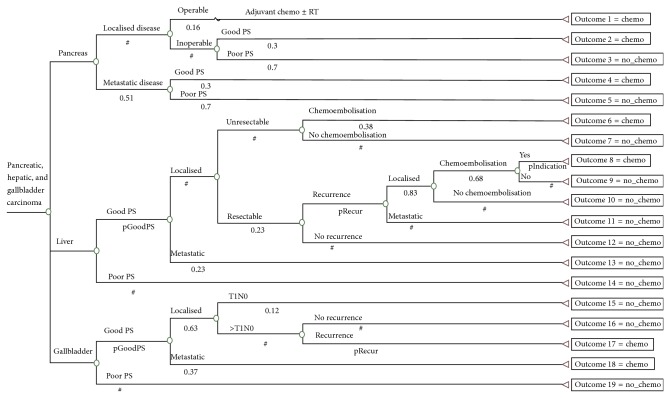
Optimal chemotherapy utilisation tree for pancreatic, liver, and gallbladder cancers.

The optimal chemotherapy utilisation trees constructed for the upper gastrointestinal cancers studied are shown in Figures 1 and 2. Each branch of the chemotherapy utilisation trees represents an important tumour or patient-related attribute that affects the chemotherapy decision. Each terminal branch of the tree shows whether or not chemotherapy is indicated for each of the clinical scenarios. The branches that end in 1 indicate that chemotherapy is recommended and the branches that end in 0 indicate that chemotherapy is not recommended for that group. The description of the attributes is located above each branch of the utilisation tree with corresponding proportion of the population with that attribute located below that branch.

There were 31 possible outcomes in the chemotherapy utilisation trees generated for the upper gastrointestinal cancers studied. Table 1 lists the 15 possible outcomes for which chemotherapy was indicated (4 for oesophageal cancer, 4 for gastric cancer, 3 for pancreatic cancer, 2 for primary liver cancer, and 2 for gallbladder cancer). The optimal chemotherapy utilisation rates calculated were 79% for oesophageal cancer, 83% for gastric cancer, 35% for pancreatic cancer, 80% for gallbladder cancer, and 27% for primary liver cancer.

### 3.1. Sensitivity Analysis

There were nine instances where the incidence data differed by more than 10% or the indications for chemotherapy were controversial. Sensitivity analysis was performed to assess the effect of these data uncertainties on the optimal chemotherapy utilisation rate for the relevant tumour site. For oesophageal cancer, the variables included the resectability rates of localised disease (0.54–0.69), the proportion of good performance status patients (0.80–0.91), and if preoperative chemoradiation treatment was indicated for patients with localised resectable oesophageal cancer. There were three uncertain variables for primary liver cancer: the proportion of good performance status patients (0.77–0.90), the recurrence rates following curative hepatectomy (0.67–0.80), and whether chemoembolisation was indicated for isolated intrahepatic recurrence following previous surgical treatment.

The remaining three uncertain variables were the proportion of good performance status patients for gastric cancer (0.76–0.89) and gallbladder cancer (0.75–0.89) and the recurrence rates of locoregional gallbladder cancer following surgical resection (0.85–0.95). No sensitivity analysis was required for the pancreatic cancer utilisation tree as the incidence data obtained did not differ by more than 10%.

Univariate sensitivity analysis shows that if preoperative chemoradiation was indicated for localised resectable oesophageal cancer, the optimal chemotherapy utilisation rate would rise from 79% to 91%. For primary liver cancer, if chemoembolisation was not indicated for intrahepatic recurrence following surgery, the optimal utilisation rate would fall from 27% to 20%. The ranges of the optimal chemotherapy utilisation rates were 69%–91% for oesophageal cancer, 76%–89% for gastric cancer, 67%–80% for gallbladder cancer, and 20%–27% for primary liver cancer.

### 3.2. Comparison with Actual Practice

Actual chemotherapy utilisation rates in upper gastrointestinal tumours have been published by the United States National Cancer Database (NCDB) [5, 8–11], United Kingdom Northern and Yorkshire Cancer Registry and Information Service (NYCRIS) [16], and Swedish Council of Technology Assessment in Health Care [12]. These studies reported the actual utilisation rates for the first course of treatment received (defined as treatment received within the first six months of diagnosis).

Overall, the actual chemotherapy utilisation rates reported for the upper gastrointestinal cancers studied were substantially lower than the estimated optimal rates with the exception of primary liver cancer (see Table 3). In the United States, the actual chemotherapy utilisation rate (first course of treatment) for primary liver cancer was the same as the optimal rate of 22% [5]. However, the actual utilisation of chemotherapy in primary liver cancer in Japan of 33% was much higher than the optimal rate [6]. The largest discrepancies between the optimal and actual chemotherapy utilisation rates were seen in the gastric cancer population, where the difference was 49% to 73% [9, 12, 16].

The utilisation rate of chemotherapy in the United Kingdom Northern and Yorkshire Cancer Registry for oesophageal, gastric, and pancreatic cancers was approximately half of the United States [9, 11, 13, 16]. The chemotherapy utilisation rates for gastric and pancreatic cancers in Sweden were also lower than the estimated optimal rates found in this study [12].

## 4. Discussion

Based on the best available evidence, we estimated the benchmarks for the optimal chemotherapy utilisation rates at 79% for oesophageal cancer, 83% for gastric cancer, 35% for pancreatic cancer, 80% for gallbladder cancer, and 27% for primary liver cancer. We found that the actual chemotherapy utilisation rates were well below the benchmarks for each of the corresponding upper gastrointestinal tumour sites studied (with the exception of primary liver cancer).

The robustness of the estimated optimal utilisation rates calculated in our model was dependent on two factors: whether the indications of chemotherapy were uniformly recommended by the guidelines and the quality of the incidence data used to define the proportion of tumour and patient attributes. The lack of available performance status data (except for pancreatic cancer) and controversial indications for chemotherapy treatment were the main contributors to the range of optimal chemotherapy utilisation rates seen when sensitivity analyses were performed.

We identified two controversial indications for chemotherapy in this study. These were whether patients with resectable oesophageal cancer should receive preoperative chemoradiation and if patients with isolated intrahepatic recurrence following hepatectomy for primary liver cancer should be treated with chemoembolisation. Several meta-analyses have concluded that the trimodality approach in patients with resectable oesophageal cancer significantly improves short term (2- or 3-year) survival when compared to surgery alone [56–59]. However, the clinical practice guidelines [18, 23, 27, 30] do not recommend preoperative chemoradiation for patients with resectable oesophageal cancer due to concerns with increased treatment-related mortality [57] and lack of longer-term followup data. Sensitivity analysis showed that if preoperative chemoradiation for oesophageal cancer was indicated, the optimal chemotherapy utilisation rate in oesophageal cancer would rise from 79% to 91%. Regardless of this controversial indication, the proportion of patients with oesophageal cancer treated with chemotherapy in the NCDB (38%–52%) and NYCRIS (15%) remains well below the optimal rate of 79% (range 69%–91%).

Chemoembolisation for patients with isolated intrahepatic recurrence of hepatocellular carcinoma following hepatectomy was recommended as a treatment option by the National Cancer Institute PDQ [25]. This recommendation was based on a small case series showing that patients with recurrent isolated intrahepatic hepatocellular carcinoma treated with chemoembolisation had 5-year survival of 14% to 20% [51, 52]. If chemoembolisation were not indicated for intrahepatic recurrence of hepatocellular carcinoma following surgery, sensitivity analysis indicated that the optimal chemotherapy utilisation rate in hepatocellular carcinoma would fall from 27% to 20%. The United States NDCB [5] reported that the proportion of patients with primary liver cancer who received chemotherapy has declined over time from 37% (1985-1986) to 22% (1995-1996), which is within the range of the estimated optimal rate.

The largest discrepancy between the optimal and actual chemotherapy utilisation rate was seen in the gastric cancer population, where the difference was 49%, 69%, and 63%–73% in the United States, United Kingdom, and Sweden, respectively [9, 12, 16]. This apparent disparity is possibly related to the recent evidence that chemotherapy for resectable gastric cancer improves survival [60] and therefore recommended by the current guidelines. The actual chemotherapy utilisation rates reported above predate this evidence. In our model, this “newer” indication represents over half of those indicated for chemotherapy in the gastric cancer population.

Apart from newer indications for chemotherapy treatment that postdate the actual utilisation data, other potential reasons for the underutilisation of chemotherapy in upper gastrointestinal tumours may include underreferrals, lack of access to chemotherapy treatment facilities, patient refusal, and clinician bias, although these are not well studied. Due to lack of studies available, we were unable to address important issues on patient preferences or competing comorbidities in this study, which may affect the benchmarks estimated. How these clinical factors impact the overall decision of whether chemotherapy is given or not to potentially eligible patients with upper gastrointestinal tumours is currently unknown. Our evidence-based models are readily adaptable to changes in chemotherapy indications or epidemiological data and can incorporate these limitations in future studies (e.g., the optimal chemotherapy utilisation rate would rise from 27% to 36% for primary liver cancer should more recent indication for sorafenib be included in this population).

## 5. Conclusions

These are the first evidence-based models developed that can estimate the optimal chemotherapy utilisation rates as benchmarks that may be useful for improving the quality of cancer care in upper gastrointestinal cancers. We observed that major shortfalls between the recommended and actual use of chemotherapy for majority of these cancers currently exist, and the magnitude of these differences varied geographically. Potential treatment benefits for achieving the best local control and survival in patients with upper gastrointestinal tumours are lost when evidence from trials are not translated into clinical practice. Future studies should focus on developing strategies to close the gap between the ideal and actual cancer care delivered.

## Figures and Tables

**Table 1:** Upper gastrointestinal cancers: indications for chemotherapy, levels, and sources of evidence.

Outcome number	Clinical scenario	Treatment indicated	Level of evidence^a^	Reference(s)	Proportion of all patients with that cancer
Oesophageal cancer					
3	Oesophageal cancer, recurrence, good PS	Palliative chemotherapy	II	NCCN [18] NCI PDQ [23] BCCA [27] SIGN [32]	0.23
5	Oesophageal cancer, localised, unresectable, good PS	Radical chemoradiation	I	NCCN [18] NCI PDQ [23] BCCA [27]	0.29
6	Oesophageal cancer, metastatic, good PS	Palliative chemotherapy	II	NCCN [18] NCI PDQ [23] BCCA [27] SIGN [32]	0.27
Total proportion of patients with oesophageal cancer in whom chemotherapy is recommended					**0.79**

Gastric cancer					
7	Gastric cancer, resected stage 1A, recurrence, good PS	Palliative chemotherapy	I	NCCN [21] NCI PDQ [22] BCCA [27] CCO [35]	<0.01
9	Gastric cancer, locoregional, resectable, higher than stage 1A, good PS	Postoperative chemoradiation or perioperative chemotherapy	II	NCCN [21] NCI PDQ [22] BCCA [27] SIGN [32]	0.42
10	Gastric cancer, locoregional disease, unresectable	Palliative chemotherapy	I	NCCN [21] NCI PDQ [22] BCCA [27] SIGN [32]	0.15
11	Gastric cancer, metastatic, good PS	Palliative chemotherapy	I	NCCN [21] NCI PDQ [22] BCCA [27] SIGN [32]	0.26
Total proportion of patients with gastric cancer in whom chemotherapy is recommended					**0.83**

Pancreatic cancer					
1	Pancreatic cancer, localised, operable	Palliative chemotherapy	I	NCCN [19] NCI PDQ [24] BCCA [35] CCO [28]	0.08
2	Pancreatic cancer, localised, inoperable, good PS	Palliative chemotherapy	II	NCCN [19] NCI PDQ [24] BCCA [35] CCO [28]	0.12
4	Pancreatic cancer, metastatic, good PS	Palliative chemotherapy	II	NCCN [19] NCI PDQ [24] BCCA [35] CCO [28]	0.15
Total proportion of patients with pancreatic cancer in whom chemotherapy is recommended					**0.35**

Hepatocellular cancer					
6	Liver cancer, localised, unresectable, good PS	Chemoembolization	I	NCCN [20] NCI PDQ [25] BCCA [27]	0.20
8	Liver cancer, localised, resected, intrahepatic recurrence, good PS	Chemoembolization	IV	NCI PDQ [25]	0.07
Total proportion of patients with hepatocellular cancer in whom chemotherapy is recommended					**0.27**

Gallbladder cancer					
17	Gallbladder cancer, locoregional, recurrence, good PS	Palliative chemotherapy	III	NCCN [20] NCI PDQ [26] BCCA [27] CCO [31]	0.47
18	Gallbladder cancer, metastatic, good PS	Palliative chemotherapy	III	NCCN [20] NCI PDQ [26] BCCA [27] CCO [31]	0.33
Total proportion of patients with gallbladder cancer in whom chemotherapy is recommended					**0.80**

PS: performance status, NCCN: National Comprehensive Cancer Network, NCI PDQ: National Cancer Institute Physicians Data Query, BCCA: British Columbia Cancer Agency, CCO: Cancer Care Ontario, and SIGN: Scottish Intercollegiate Guidelines Network.

^a^Levels of evidence: level I: evidence obtained from a systematic review of all relevant randomised controlled trials; level II: evidence obtained from at least one properly designed randomised controlled trial; level III: evidence obtained from well-designed controlled trials without randomisation (these include trials with “pseudorandomisation” where a flawed randomisation method was used (e.g., alternate allocation of treatments) or comparative studies with either comparative or historical controls); level IV: evidence obtained from case series. Taken from the National Health and Medical Research Council (NHMRC) hierarchy of levels of evidence [33].

**Table 2:** The incidence of attributes used to define indications for chemotherapy.

Population or subpopulation of interest	Attribute	Proportion of populations with this attribute	Quality of information^a^	References
Oesophageal cancer				
All registry cancers	Oesophageal cancer	0.01	*α*	AIHW [3]
Oesophageal cancer	Good PS	0.80–0.91	*α* *δ*	AIHW [3] NSW Population Health Survey [36]
Oesophageal cancer	Metastatic disease	0.26	*β*	NSW Cancer Registry [37]
Oesophageal cancer, localised disease	Resectable	0.54–0.69	*θ*	Enzinger and Mayer [38]
Oesophageal cancer, localised disease, operable	Recurrence	0.66	*θ*	Burmeister et al. [39]

Gastric cancer				
All registry cancers	Gastric cancer	0.02	*α*	AIHW [3]
Gastric cancer	Good PS	0.76–0.89	*α* *δ*	AIHW [3] NSW Population Health Survey [36]
Gastric cancer	Metastatic disease	0.29	*β*	NSW Cancer Registry [40]
Gastric cancer, locoregional disease	Resectable	0.77	*δ*	Wanebo et al. [41]
Gastric cancer, resected stage 1A	Recurrence	0.05 0.04	*ζ* *ζ*	Yoo et al. [42] Sano et al. [43]

Pancreatic cancer				
All registry cancers	Pancreatic cancer	0.02	*α*	AIHW [3]
Pancreatic cancer	Below 80 years old	0.72	*α*	AIHW [3]
Pancreatic cancer	Metastatic disease	0.51	*β*	NSW Cancer Registry [44]
Pancreatic cancer, localised disease	Operable	0.16	*γ*	Janes Jr. et al. [45]
Pancreatic cancer, advanced disease	Good PS	0.30	*λ*	Brasiunas et al. [46]

Primary liver cancer				
All registry cancers	Liver cancer	0.01	*α* *β*	AIHW [3] NSW Cancer Registry [40]
Liver cancer	Good PS	0.90	*α* *δ*	AIHW [3] NSW Population Health Survey [36]
Liver cancer	Metastatic disease	0.23	*β*	NSW Cancer Registry [40]
Liver cancer, localised disease	Resectable	0.23	*γ*	NCDB [5]
Liver cancer, localised disease, unresectable	Suitable for chemoembolisation	0.38	*ε*	Llovet et al. [47]
Liver cancer, localised disease, resectable	Recurrence	0.2–0.33	*θ*	Jaeck et al. [48]
Liver cancer, localised disease, resectable, recurrence	Intrahepatic recurrence only	0.83 0.74	*ζ* *ζ*	Yang et al. [49] Cha et al. [50]
Liver cancer, localised disease, resectable, intrahepatic recurrence only	Suitable for chemoembolisation	0.68 0.58	*λ* *λ*	Poon et al. [51] Takayasu et al. [52]

Gallbladder cancer				
All registry cancers	Gallbladder cancer	0.01	*α*	AIHW [3]
Gallbladder cancer	Good PS	0.68	*α* *δ*	AIHW [3] NSW Population Health Survey [36]
Gallbladder cancer	Locoregional, recurrence	0.85–0.95	*γ*	NCDB [8]
Gallbladder cancer	Metastatic disease	0.37	*γ*	SEER [53]
Gallbladder cancer	Stage IA	0.12	*γ*	NCDB [8]

AIHW: Australian Institute of Health and Welfare, NSW: New South Wales, SEER: Surveillance Epidemiology and End Results, NCDB: National Cancer Database, and PS: performance status.

^a^Hierarchy for epidemiological data: *α*: Australian National Epidemiological data; *β*: Australian State Cancer Registry; *γ*: epidemiological databases from other large international groups (e.g., SEER); *δ*: results from reports of a random sample from a population; *ε*: comprehensive multi-institutional database; *ζ*: comprehensive single-institutional database; *θ*: multi-institutional reports on selected groups (e.g., multi-institutional clinical trials); *λ*: single-institutional reports on selected groups of cases; *μ*: expert opinion (adapted from Delaney et al. [34]).

**Table 3:** Comparison of optimal and actual chemotherapy utilisation rates for upper gastrointestinal cancers.

Tumour site	Optimal chemotherapy utilisation rate (%)		Actual chemotherapy utilisation rate (%)		
	Any time	First course treatment	United States NCDB [1, 8–11, 13]	United Kingdom NYCRIS [16]	Sweden [12]
Oesophagus	79	57	38–52	15	NR
Stomach	83	83	34	14	10–20
Pancreas	35	35	19–37	11	20–30
Primary liver	27	22	22–37	NR	NR
Gallbladder	80	33	22	NR	NR

NCDB: National Cancer Database, NYCRIS: Northern and Yorkshire Cancer Registry, and NR: not reported.
